# Relationship Between Integrity of the Corpus Callosum and Bimanual Coordination in Children With Unilateral Spastic Cerebral Palsy

**DOI:** 10.3389/fnhum.2019.00334

**Published:** 2019-09-24

**Authors:** Ya-Ching Hung, Maxime T. Robert, Kathleen M. Friel, Andrew M. Gordon

**Affiliations:** ^1^Department of Family, Nutrition, and Exercise Sciences, Queens College, The City University of New York, New York, NY, United States; ^2^Department of Biobehavioral Sciences, Teachers College, Columbia University, New York, NY, United States; ^3^Burke Neurological Institute, Weill Cornell Medicine, White Plains, NY, United States

**Keywords:** corpus callosum, pediatric, diffusion MRI, kinematics, upper extremity, cerebral palsy

## Abstract

Children with unilateral spastic cerebral palsy (USCP) have shown impaired bimanual coordination. The corpus callosum (CC) connects the two hemispheres and is critical for tasks that require inter-hemisphere communication. The relationship between the functional bimanual coordination impairments and structural integrity of the CC is unclear. We hypothesized that better integrity of the CC would relate to better bimanual coordination performance during a kinematic bimanual drawer-opening task. Thirty-nine children with USCP (Age: 6–17 years old; MACS levels: I-III) participated in the study. Measurement of the CC integrity was performed using diffusion tensor imaging. The CC was measured as a whole and was also divided into three regions: genu, midbody, and splenium. Fractional anisotropy, axial diffusivity (AD), radial diffusivity, mean diffusivity, number of voxels, and number of streamlines were evaluated in whole and within each region of the CC. 3-D kinematic analyses of bimanual coordination were also assessed while children performed the bimanual task. There were negative correlations between bimanual coordination measures of total movement time and AD of whole CC (*p* = 0.037), number of streamlines and voxels of splenium (*p* = 0.038, 0.032, respectively); goal synchronization and AD of whole CC (*p* = 0.04), and number of streamlines and voxels of splenium (*p* = 0.001, 0.01, respectively). The current results highlight the possible connection between the integrity of the CC, especially between the splenium region and temporal bimanual coordination performance for children with USCP.

## Introduction

Children with unilateral spastic cerebral palsy (USCP) have early brain damage that leads to various motor and sensory impairments particularly on their more-affected side, such as slower movement and impaired hand grasping control (e.g., Eliasson et al., 1995; Forssberg et al., 1999; Gordon et al., 2003; Steenbergen et al., 2008). Severity of impairments on the more-affected hand has been showed to be associated with poorer bimanual coordination (Hung et al., 2004). Studies of children with USCP also indicated impaired bimanual hand function performance using clinical tests [e.g., assisting hand assessment (AHA)] and kinematic tasks (e.g., Utley and Sugden, 1998; Hung et al., 2004, 2010; Utley et al., 2004; Gaillard et al., 2018). Kinematic analysis showed that bimanual coordination impairments are associated with reduced temporal coordination between the two hands during symmetric and asymmetric bimanual tasks in children with USCP (e.g., Utley and Sugden, 1998; Hung et al., 2004, 2010; Utley et al., 2004). All of these movement impairments affect their ability to perform daily functional activities and their independence (e.g., Lee, 2017).

It is essential to better understand the connection between brain white matter integrity and movement performances for children with USCP to better predict their prognosis and to develop specific effective treatments. Reid et al. (2015) indicated that severe white matter loss of both hemispheres and the corpus callosum (CC) measured by magnetic resonance imaging (MRI) is associated with poor gross motor function [gross motor function classification (GMFCS)]. They further suggested that MRI may not be sensitive enough to detect microstructural impairments such as connectivity within the white matter. More advanced neuroimaging techniques such as diffusion tensor imaging (DTI) provides a more sensitive measure of white matter microstructure allowing the reconstruction of neuronal pathways (Mori et al., 1999). Diffusion parameters such as number of streamlines, number of voxels, and fractional anisotropy (FA, directional preference of white matter water molecular diffusion measure; higher value reflecting better axonal integrity) are often reported in studies on individuals with USCP (e.g., Thomas et al., 2005). Children with USCP were found to have decreased fiber count on the corticobulbar tract, increased mean diffusivity (MD, degree of restriction to diffusion of white matter water molecules irrespective to direction; lower value reflecting better axonal integrity) and decreased FA on the primary white matter lesion site (e.g., Thomas et al., 2005). Cortico-spinal tract diffusion properties (lower FA and higher MD) were also shown to be correlated with the severity of movement impairments (Kuczynski et al., 2018).

Using diffusion tensor imaging, Weinstein et al. (2014) showed that poorer integrity of the CC is associated with more-severe unimanual and bimanual hand function evaluated by clinical measures of AHA and children’s hand experience questionnaire. CC is the main pathway between the hemispheres. A larger CC size has been shown to be related to better motor performance in children with periventricular leukomalacia and children born prematurely (Davatzikos et al., 2003; Rademaker et al., 2004; Pannek et al., 2014). The communication role between the hemispheres of the CC is especially important for motor control involving bimanual coordination (see review, Bloom and Hynd, 2005). The less-affected hand was suggested to compensate for the more affected hand during some bimanual tasks for children with USCP (e.g., Utley and Sugden, 1998; Hung et al., 2004, 2010; Utley et al., 2004). Such compensatory strategies between the two hands would depend on interhemispheric communication and should correlate to microstructure impairments of the CC.

In our previous studies, children with USCP showed impaired temporal bimanual coordination during a functional bimanual drawer task using kinematic analysis (Hung et al., 2004, 2010). The test was sensitive in order to identify differences in improvements after intensive unimanual and bimanual interventions (Hung et al., 2011, 2017a). Thus, this drawer task can be an ideal test to evaluate the connection between the integrity of the CC and kinematic bimanual performance in children with USCP.

Our objective is to explore the relationship between the integrity of the CC and the kinematics of bimanual performance during a functional bimanual drawer task in children with USCP. We hypothesized that DTI parameters of the CC will be correlated with temporal bimanual coordination measures.

## Materials and Methods

### Participants

A subset of thirty-nine children with USCP (20 males, 19 females, age 6–17 years, MACS I-III, Table 1) from a prior randomized control trial (RCT, NCT02918890) were recruited over 4 years (2014–2017) to participate in this study. The RCT recruited participants from the website^1^, clinics in the NYC area, and online support groups. All potential participants were screened by an on-site physical examination or a videotaped examination by their physical/occupational therapist first. The inclusion criteria were selected based on our prior USCP studies: (1) attending a mainstream school, (2) the ability to follow instructions during screening, (3) the ability to lift the more affected arm 15 cm above a table and grasp light objects, and (4) the ability to complete all testing. The exclusion criteria were: (1) botulinum toxin in the upper extremity within the last 6 months, (2) other health problems unassociated with CP, (3) current/unstable seizures, (4) visual problems interfering with testing, and (5) surgery on the more affected hand within 1 year. Informed consents were obtained from all the participants and guardians. The current study was approved by the University Institutional Review Board.

**TABLE 1:** Participant characteristics.

**Affected Side**	**Right (*n* = 15)**	**Left (*n* = 24)**
Mean Age (*SD*) y,m	9,9 (3,2)	9,3 (3,3)
Age range y	6–17	6–17
Gender		
Male	6	14
Female	9	10
MACS		
I	4	8
II	8	12
III	3	4
AHA (*SD*) AHA units	55.60 (7.43)	56.33 (10.11)

*SD, standard deviation; MACS, manual ability classification system for individuals with cerebral palsy; AHA, assisting hand assessment.*

### Procedures

All participants were part of a RCT to have intensive upper extremity training. Neuroimaging, bimanual coordination using a 3D motion capture system, and AHA score were assessed before their training to elucidate the mechanisms between the integrity of the CC and bimanual coordination.

### MRI Data Acquisition

Diffusion MRI was acquired on all 39 children. DTI was used to reconstruct the interhemispheric connections of the CC. The MRI protocol was performed on a 3T Scanner (Siemens Magnetom Trio, Citigroup Biomedical Imaging Center, Weill Cornell Medical College). A total of 75 slices were acquired (matrix 112 × 112, FOV = 224 mm, 65 directions, b-value = 800 s/mm^2^, TR = 9000 ms, and TE = 83 ms). The participants were positioned in a supine position with padding around the head to minimize the movement and reduce noise. The participants were not physically constrained nor received any sedation.

### MRI Data Analysis

Diffusion tensor imaging analysis was performed using DTI Studio (John Hopkins University, Baltimore, MD, United States), which included FA, vector maps, and color-coded maps. An image was first created to mask the background noise at the threshold of 30 dB, using standard linear regression for tensor calculation. Images containing movement artifacts were excluded by visually inspecting the original images using the apparent diffusion constant function (Jiang et al., 2006). Reconstruction of the interhemispheric connections was done using the Continuous Tracking method (Mori et al., 1999). Fiber tracking started <0.15 and was terminated if the tract turning angle was >70.

Regions of interest were determined using anatomical location through orientation-based color-coding maps by hand. The CC was segmented into the following three segments based on the Witelson parcelation scheme: genu, midbody, and splenium (Witelson, 1989).

The FA, axial diffusivity (AD, reflecting axonal growth and injury), radial diffusivity (RD, indicating myelination and demyelination processes), and MD, number of streamlines and voxels were calculated. Data was analyzed by a trained researcher (second author) blinded to the kinematic results of participants. To ensure good reliability of the DTI findings, we evaluated both inter-rater and intra-rater variability. Good to excellent reliability was found: coefficient ranging of inter-rater from 0.816 (CI 0.079–0.963) to 0.979 (CI 0.896–0.996) and intra-rater from 0.746 (CI −0.267–0.949) to 0.988 (CI 0.940–0.998).

### Kinematic Bimanual Coordination Testing

Participants were instructed to open a spring-loaded drawer (load 0.3 kg) with the less affected (drawer hand) and to insert their more affected hand (task hand) in the drawer to activate a light switch (14 cm × 10 cm) while seated. Participants were seated in front of the table (15 cm from the trunk) with both elbows flexed at right angles, and hands placed 30 cm apart at the edge of the table initially. The drawer (15 cm × 15 cm) equipped with a loop handle (9 cm in length and 3 cm in depth) and was placed at midline 30 cm from the edge of the table from the subject.

The drawer task was performed at a self-selected speed while 3-D kinematic data were collected with eight infrared cameras using Workstation 4.6 (VICON, Denver, CO, United States). Each trial ended after the subject activated the light switch inside the drawer. Total of five trials were collected after two practice trials. Seven reflective markers were placed on bilateral shoulder (acromion process), elbow (lateral epicondyle), wrist (ulnar styloid process), and spinous process of the seventh cervical vertebra (C7) of the participants. Calibration was performed on the space with a set external x (medial-lateral axis), y (anterior-posterior axis), and z (vertical axis) coordinates. The digitizing rate was 120 Hz. All digitized signals were processed using a low pass digital filter with a cutoff frequency of 6 Hz.

The velocity onset/offset threshold was set at a criterion of 5% peak velocity of the wrist tangential velocity. The onset of hand movement was defined when the wrist tangential velocity exceeded the criterion and constantly moved forward thereafter. The end of drawer opening (offset of drawer hand) was defined as the velocity falling below the criterion. The offset of the more affected hand was defined as the time when the wrist tangential velocity fell below the velocity criterion or when the light switch inside the drawer was activated.

The overall task completion time was defined by the time between the onset of the drawer hand and the offset of the task hand movements. The goal synchronization of the two hands was measured by the time difference between the offset of the two hands (the drawer hand fully opening the drawer and the task hand reaching inside the drawer). The normalized movement overlap time was calculated by the overlapping movement time of the two hands, as a percentage of the total task completion time. These three temporal measures were used to evaluate bimanual coordination performance. 3-D displacement of C7 marker was also calculated to indicate the trunk motion during task performance.

### Quality of Bimanual Hand Use

The assisting hand assessment (AHA, version 5.0) quantifies the effectiveness to which children with unilateral impairments utilize their more affected hand to assist various bimanual play activities and has excellent validity/reliability (Holmefur et al., 2007; Krumlinde-Sundholm et al., 2007). All participants were videotaped during testing and were scored offsite by an experienced blinded evaluator. Data were reported in logit-based units.

### Statistical Analysis

Statistical analysis was performed using SPSS (version 23, Statistical Production and Service Solutions, Chicago, IL, United States). To better understand the relationship between the DTI parameters and the kinematic measures of bimanual coordination, partial Spearman’s correlations controlling for age was used. To explore the connection between the current temporal bimanual coordination measures and trunk control with clinical measure of AHA, a separate partial Spearman’s correlations controlling for age was carried out. In order to examine the possible effects of the side of hemiplegia on all the kinematic measures and AHA, a simple *t*-test with either side of hemiplegia (left or right) was performed. All of the data were approximately normally distributed (Shapiro-Wilk test). Significance was set at *p* < 0.05.

## Results

### Temporal Bimanual Coordination and DTI Parameters

**FIGURE 1:**
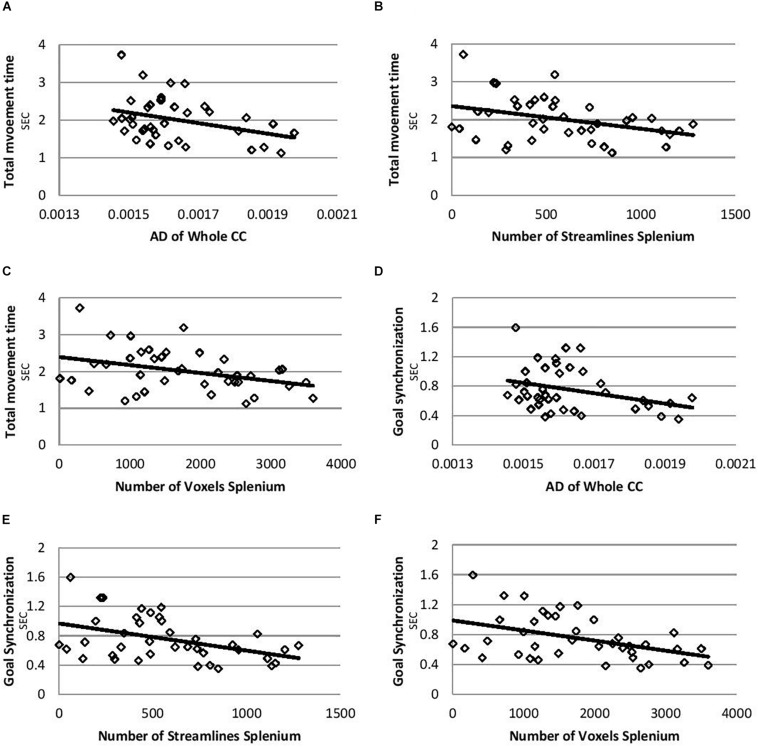
Correlation plots for total movement time and goal synchronization. **(A)** Correlation between total movement time and AD of whole CC. **(B)** Correlation between total movement time and number of streamlines of splenium. **(C)** Correlation between total movement time and number of voxels of splenium. **(D)** Correlation between goal synchronization and AD of whole CC. **(E)** Correlation between goal synchronization and number of streamlines of splenium. **(F)** Correlation between goal synchronization and number of voxels of splenium. AD, axial diffusivity; CC, corpus callosum.

Correlations controlling for age between temporal bimanual coordination and DTI parameters are shown in Table 2. The total movement time was negatively correlated with AD of the whole CC (Figure 1A, *r* = −0.33, *p* = 0.037), number of streamlines of splenium (Figure 1B, *r* = −0.34, *p* = 0.038), and number of voxels of the splenium (Figure 1C, *r* = −0.35, *p* = 0.032). Goal synchronization differences were negatively correlated with AD of the whole CC (Figure 1D, *r* = −0.33, *p* = 0.04), number of streamlines of the splenium (Figure 1E, *r* = −0.40, *p* = 0.01), and number of splenium voxels (Figure 1F, *r* = −0.42, *p* = 0.01). No correlation was observed for the genu and midbody for any measure.

**TABLE 2:** Correlation analyses between temporal coordination measures and DTI variables.

**Region**	**DTI**	**Total**		**Goal**		**Normalized**	
	**variables**	**movement**		**synchronization**		**movement**	
		**time**				**overlap**	
		***R***	***p* value**	***R***	***p* value**	***R***	***p* value**
Whole CC	FA	–0.003	0.98	–0.07	0.69	–0.15	0.39
	Streamlines	–0.12	0.47	–0.23	0.17	–0.09	0.61
	Voxels	–0.14	0.39	–0.26	0.12	–0.07	0.70
	AD	–0.33	0.037^∗^	–0.33	0.04^∗^	0.09	0.61
	RD	0.18	0.28	0.22	0.18	–0.02	0.93
	MD	–0.24	0.14	–0.23	0.17	–0.05	0.77
Genu CC	FA	–0.033	0.84	–0.03	0.87	–0.08	0.62
	Streamlines	–0.20	0.23	–0.27	0.11	0.06	0.72
	Voxels	–0.19	0.26	–0.28	0.09	0.10	0.56
	AD	–0.29	0.075	–0.28	0.09	–0.15	0.38
	RD	–0.14	0.40	–0.14	0.42	–0.06	0.72
	MD	–0.23	0.16	–0.22	0.18	–0.11	0.51
Midbody	FA	–0.02	0.92	–0.03	0.87	–0.03	0.85
CC	Streamlines	–0.10	0.55	–0.21	0.20	0.09	0.56
	Voxels	–0.11	0.52	–0.22	0.19	0.11	0.51
	AD	–0.30	0.064	–0.27	0.11	–0.18	0.29
	RD	–0.08	0.65	–0.05	0.76	–0.06	0.70
	MD	–0.21	0.22	–0.17	0.30	–0.13	0.44
Splenium	FA	–0.08	0.63	–0.14	0.40	–0.02	0.93
CC	Streamlines	–0.34	0.038^∗^	–0.40	0.01^∗^	0.07	0.68
	Voxels	–0.35	0.032^∗^	–0.42	0.01^∗^	0.01	0.95
	AD	–0.17	0.40	–0.19	0.25	–0.03	0.87
	RD	0.004	0.98	0.04	0.82	0.01	0.93
	MD	0.08	0.62	–0.07	0.67	–0.01	0.98

*CC, corpus callosum; DTI, diffusion tensor imaging; FA, fractional anisotropy; AD, axial diffusivity; RD, radial diffusivity; MD, mean diffusivity; Streamlines, number of streamlines; Voxels, number of voxels; ^∗^, significant correlation.*

### Clinical AHA Test and Kinematic Bimanual Coordination Variables

Table 3 shows the correlation summary of AHA scores with temporal bimanual coordination and trunk involvement. There were significant negative correlations between AHA scores and most kinematic valuables; i.e., better bimanual coordination was associated with higher AHA scores (see Table 3, except normalized overlap movement). The three temporal bimanual coordination variables were significantly correlated to one another (total time and goal synchronization: *r* = 0.92 *p* = 0.001; total time and normalized overlap *r* = −0.42, *p* = 0.009; goal synchronization and normalized overlap: *r* = −0.55, *p* = 0.001), but not with trunk control. Trunk control was negatively correlated with AHA score (*r* = −0.43, *p* = 0.008).

**TABLE 3:** Correlation analyses between AHA scores and kinematic measures.

	**AHA**		**Total**		**Goal**		**Normalized**	
			**movement time**		**synchronization**		**movement overlap**	
	***R***	***p* value**	***R***	***p* value**	***R***	***p* value**	***R***	***p* value**
Total movement time	–0.33	0.041^∗^						
Goal synchronization	–0.35	0.03^∗^	0.92	0.001^∗^				
Normalized movement overlap	–0.016	0.33	–0.42	0.009^∗^	–0.55	0.001^∗^		
Trunk control	–0.33	0.042^∗^	0.74	0.055	0.63	0.08	0.20	0.21

*AHA, assisting hand assessment; ^∗^, significant correlation.*

### Effects of Hemiplegia Side

No significant findings of hemiplegic side effects on temporal bimanual coordination measures, trunk control, or AHA score were found.

## Discussion

This study investigated the relationship between the CC diffusion properties and bimanual coordination performance during a functional bimanual drawer task in children with USCP. As we hypothesized, the integrity of the CC was correlated with temporal bimanual coordination performance for children with USCP. AD of the whole CC and the splenium region were correlated with bimanual coordination performance. Genu and midbody diffusion properties were not related to any measure. There were also significant correlations between clinical bimanual AHA scores and bimanual coordination and trunk control measures.

### Correlation Between Bimanual Coordination and Integrity of the Splenium

There were significant negative correlations between total movement time and goal synchronization measures and AD of the whole CC, number of streamlines of the splenium, and the number of voxels of the splenium. Shorter total movement time and goal synchronization time differences both indicated better temporal bimanual coordination during the current task. Children with USCP showed impaired temporal bimanual coordination during a drawer task similar to the current one when compared with typically developed children (Hung et al., 2004, 2010). They had longer total movement time, reduced goal synchronization (longer goal accomplished time differences between the two hands), and reduced normalized movement overlap (Hung et al., 2004, 2010). Thus the current negative correlations indicated better bimanual control correlated with integrity of microstructure of the CC, especially the splenium. The number of streamlines of the splenium was also correlated with AHA scores (clinical bimanual measure) for children with USCP in a previous study (Davatzikos et al., 2003).

Weinstein et al. (2014) showed only correlations between the splenium region integrity (FA, MD, and RD measures) and clinical hand function measures (questionnaire of more affected hand use during daily bimanual tasks) in children with USCP. Weinstein et al. (2014) further suggested the possible reason of their findings was the important role of the splenium region for visual-spatial control and spatial awareness of the more affected hand. Thus the number of streamlines of the splenium region that influenced bimanual coordination performance (such as current kinematic measures and AHA scores of previous study), and the quality of fibers (integrity) seemed to be connected with spatial temporal movement control of the more affected hand. Since we did not assess unimanual spatial temporal control of the more affected hand, we cannot evaluate the suggestion from Weinstein et al. (2014).

The possible reason for failing to find correlation between splenium integrity (FA, MD, and RD measures) and kinematic bimanual coordination in the current study could be the influence of developmental factors. The number of streamlines may not be affected as greatly as integrity changes of the splenium during development. Muetzel et al. (2008) evaluated the relationship between development of the CC and bimanual performances for healthy adolescents. They found that splenium FA measures correlated greatly with bimanual finger tapping performance. They did not measure number of streamlines or voxels. However, they suggested increase splenium FA in DTI studies to be related to splenium white matter volume. Muetzel et al. (2008) suggested that white matter integrity (measured by FA) in the splenium continued to develop until 18 years old for healthy adolescents. The developmental process and influence factors of such white matter integrity (such as measure of FA) for children with USCP is unclear and most likely prolonged with a different developing rate. The current study had a wide age range (6–17 years old) and was more likely to be influenced by developing factors even though the correlation findings were controlled for age statistically. The age effect of CC integrity changes between 6 and 7 years old is not likely to be the same as between 15 and 16 years old. A future study with more participants to evaluate the developmental effects of the number of streamlines and voxels, and integrity measures (control for severity) could help answer this question. The other possible reason could be that other neural pathways (e.g., Cortico-spinal tract, cerebellum) provide additional compensatory mechanisms.

In the current study, AD of the whole CC was also negatively correlated with total movement time and goal synchronization performance for children with USCP. AD was suggested to evaluate axonal integrity (Harsan et al., 2006; Sun et al., 2006). However, most microstructure studies of the CC failed to indicate significant AD findings (e.g., Weinstein et al., 2014). In a previous study that evaluated changes of white matter microstructure of the CC after after-school additional physical activity program (9 month program) for children indicated changes in FA and RD measures of the CC, but not AD values (Chaddock-Heyman et al., 2018). AD measure of the CC may be less likely to change over training or it has less sensibility to detect small changes. The previous studies only evaluated the connection between AD of the CC and clinical measures which may be less sensitive to movement coordination than kinematic analysis (Hung et al., 2011, 2017a,b; Weinstein et al., 2014; Serrien, 2017).

### AHA and Bimanual Kinematic Measures

It is interesting to see the connection between the AHA score (clinical bimanual measure) and kinematic bimanual coordination measures of total movement time, goal synchronization, and trunk control during the current drawer task. Most of the previous studies failed to find any significant correlation between the AHA and kinematic bimanual coordination measures (Hung et al., 2011, 2017a). A larger group of participants and wider age range of participants with the developmental effects may lead to significant correlation findings.

Trunk control was significantly correlated to AHA scores, but not other temporal bimanual coordination measures. Proximal trunk compensation (excessive trunk forward movement) for the reduced distal joint motion of the more affected upper extremity was shown in previous studies for children with USCP (e.g., Coluccini et al., 2007; Hung et al., 2012). Therefore, trunk control during the current task might be more related to impairments of the more affected hand than the temporal quality of bimanual movement coordination. The AHA also emphasizes how the more affected hand performs hand assists for the less affected hand during bimanual tasks. The AHA does not measure the coordination between the two hands (e.g., how less affected hand slows down to compensate for the more affected hand).

### Limitations

The current project has a wide age range of participants. This may increase the possibility of developmental influences even if we statistically tried to control for the age effect. No control group was included in the study, which could provide better age-specific comparison. We only focused on the integrity of the CC; however, there are many other regions of the brain or descending tracts that might be correlated to bimanual coordination performance as well. Multiple correlations are evaluated in our study, and may increase type I error. To avoid false significant findings due to multiple correlations, we confirmed same findings with linear regressions.

## Conclusion

The current study investigated the connection between the CC diffusion properties (assessed by DTI) and bimanual coordination performance during a functional bimanual drawer opening task for children with USCP. The integrity of the CC, specifically the splenium, was found to play a role in bimanual coordination performance. This means that the splenium might be important for temporal coordination for movement control. Future studies are required to further understand the underlying mechanisms between the microstructure of the central nervous system (e.g., connections between the different neuronal pathways and other structures) and the various movement performance. Therefore, we can potentially predict the movement impairments. It would also be important to determine the interaction of the integrity of the CC and changes in bimanual coordination following intensive therapies.

## Data Availability Statement

The datasets generated for this study are available on request to the corresponding author.

## Ethics Statement

The studies involving human participants were reviewed and approved by the Teachers College, Columbia University Burke Neurological Institute, Weill Cornell Medicine Queens College, and City University of New York. Written informed consent to participate in this study was provided by the participants’ legal guardian/next of kin.

## Author Contributions

Y-CH and MR designed and conducted the experiments. Y-CH analyzed the data and wrote the manuscript. AG and KF helped to discuss the data and edited the manuscript.

## Conflict of Interest

The authors declare that the research was conducted in the absence of any commercial or financial relationships that could be construed as a potential conflict of interest.

## Footnotes

1 http://www.tc.edu/centers/cit/
